# When Learning and Remembering Compete: A Functional MRI Study

**DOI:** 10.1371/journal.pbio.1000011

**Published:** 2009-01-13

**Authors:** Willem Huijbers, Cyriel M Pennartz, Roberto Cabeza, Sander M Daselaar

**Affiliations:** 1 Center for Neuroscience, Swammerdam Institute for Life Sciences, Faculty of Science, University of Amsterdam, Amsterdam, The Netherlands; 2 Center for Cognitive Neuroscience, Duke University, Durham, North Carolina, United States of America; 3 Department of Psychology and Neuroscience, Duke University, Durham, North Carolina, United States of America; University of California, Irvine, United States of America

## Abstract

Recent functional neuroimaging evidence suggests a bottleneck between learning new information and remembering old information. In two behavioral experiments and one functional MRI (fMRI) experiment, we tested the hypothesis that learning and remembering compete when both processes happen within a brief period of time. In the first behavioral experiment, participants intentionally remembered old words displayed in the foreground, while incidentally learning new scenes displayed in the background. In line with a memory competition, we found that remembering old information was associated with impaired learning of new information. We replicated this finding in a subsequent fMRI experiment, which showed that this behavioral effect was coupled with a suppression of learning-related activity in visual and medial temporal areas. Moreover, the fMRI experiment provided evidence that left mid-ventrolateral prefrontal cortex is involved in resolving the memory competition, possibly by facilitating rapid switching between learning and remembering. Critically, a follow-up behavioral experiment in which the background scenes were replaced with a visual target detection task provided indications that the competition between learning and remembering was not merely due to attention. This study not only provides novel insight into our capacity to learn and remember, but also clarifies the neural mechanisms underlying flexible behavior.

## Author Summary

This study provides clear evidence for a bottleneck in our memory system between learning new and remembering old information. The ability to continuously learn and remember is usually taken for granted. Virtually all interactive situations we encounter require concurrent learning and remembering. For example, normal social communication requires that we process the new information that another person is providing. While listening, we are usually already retrieving information in preparation of an appropriate reply. Other examples include driving through an unfamiliar city while interpreting familiar traffic signs, or encountering novel products during shopping while remembering what we need. Although these examples clearly illustrate the importance of the simultaneous occurrence of learning and remembering, this study shows that remembering and learning compete for resources when both processes happen within a brief period. The study also examined the neural consequences of the competition between learning and remembering using functional MRI (fMRI). In line with the behavioral competition, the neuroimaging results showed a clear suppression of learning-related brain activity as a result of concurrent remembering. Finally, the study provides evidence that a specific region in the prefrontal cortex can resolve the bottleneck, possibly by allowing rapid switching between learning and remembering

## Abstract 3

When we try to learn and remember at the same time, a bottleneck occurs within our memory system with both behavioral and neural costs.

## Introduction

We continuously learn novel events (memory encoding) and remember past events (memory retrieval): this fact is so intricately woven into the fabric of our personal lives that we easily take it for granted. Yet, this central aspect of daily life is not as straightforward as it might seem. In fact, many influential models of memory assume that encoding and retrieval cannot occur at the same time and that the two processes compete for neural resources [1–3]. In line with a competition, recent functional neuroimaging studies have indicated opposing levels of brain activity during encoding and retrieval. In particular, successful retrieval has been associated with increased activity in the posterior cingulate cortex (PCC) [4,5], whereas successful encoding has been associated with decreased activity in this same region [6–8]. Given that global activity in a particular brain region cannot increase and decrease at the same time, these findings lead to the hypothesis that successful learning and successful remembering may compete when both processes happen concurrently. In two behavioral experiments (our Behavioral Experiments 1 and 2) and one functional MRI (fMRI) experiment, we investigated the behavioral and neural consequences of this potential bottleneck in the human memory system.

The study used a novel paradigm that forces encoding and retrieval to happen within a brief period of time. The experimental task involves three phases: a *word encoding phase* (Figure 1A), a *word retrieval/scene encoding phase* (Figure 1B), and a *scene retrieval phase* (Figure 1C). During the *word encoding phase*, participants rapidly encode words by processing their meaning (living/nonliving decisions). During the *word retrieval/scene encoding phase*, participants perform an old/new word recognition task including words presented at the word encoding phase intermixed with new words. The key difference with a standard old/new word recognition test is that, while recognizing the words, participants incidentally encode spatial scenes that are presented in the background. To ensure simultaneous encoding and retrieval, participants are allowed maximally 1.2 s to make the recognition judgment, and both the word and scene disappear immediately after the recognition response is made. Subsequently, visually masking noise is presented to avoid further visual processing. Participants are instructed to perform the retrieval task as quickly as possible without making errors. During the *scene retrieval phase*, learning of the spatial scenes is measured with a standard old/new recognition test.

**Figure 1:**
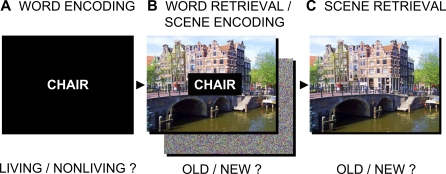
Word Retrieval/Scene Encoding Task The experimental task involved three phases: *word encoding*, *word retrieval/scene encoding*, and *scene retrieval*. (A) During *word encoding*, participants studied words while making semantic (living/nonliving) decisions about the study items. (B) During *word retrieval/scene encoding*, participants performed an old/new word recognition task including words presented at *word encoding* as well as words that were not seen at study. Simultaneously, spatial scenes were presented in the background (C) During *scene retrieval*, encoding of these spatial scenes was tested during a picture recognition task.

When combined with fMRI, this paradigm allows the measurement of both the activity associated with successful retrieval (old words classified as old versus new) and with successful encoding (scenes subsequently remembered versus forgotten) during one single task. As a result, there are four relevant trial types: word retrieval is unsuccessful but scene encoding is successful (R–E+), both word recognition and scene encoding are unsuccessful (R–E–), both word recognition and scene encoding are successful (R+E+), and finally, word recognition is successful but scene encoding is unsuccessful (R+E–). Critically, the paradigm is not simply measuring potential interference between viewing scenes and making recognition responses, but specifically measures interference between successful *encoding* and successful *retrieval*. Potential interference from perceptual or motor processes is subtracted out, because all trials have scenes in the background and all involve recognition responses.

This study tested three predictions. First, as a behavioral consequence of the bottleneck, we predicted that learning and concurrent remembering should compete. In other words, we expected that encoding of the spatial scenes would be significantly poorer when simultaneous word retrieval was successful compared with when retrieval was unsuccessful. Second, based on fMRI studies of encoding and retrieval indicating opposing levels of brain activity in PCC [4–8], we predicted that activity in this region would show an interaction between memory phase (encoding versus retrieval) and outcome (successful versus unsuccessful). We predicted that PCC activity should be highest during successful retrieval and unsuccessful encoding, and lowest during unsuccessful retrieval and successful encoding. Finally, we predicted that the behavioral effect would be coupled with suppression of brain activity in areas associated with successful encoding of spatial scenes, including the visual cortex and medial temporal lobe (MTL).

## Results

### Behavioral Experiment 1: Word Retrieval and Scene Encoding

Overall memory performance as defined by d-prime was 0.95 ± 0.13 for the word recognition and 0.70 ± 0.07 for recognition of the spatial scenes. In addition, the response bias criterion C [9] indicated that participants maintained a generally conservative response criterion during both word (C = 0.52 ± 0.08; *t* = 6.94, *p* = 0.0001) and scene (C = 1.16 ± 0.08; *t* = 15.3, *p* < 0.0001) retrieval. This positive response bias was conform our specific instructions to respond only “old” when certain. Response times (RTs) during the word recognition task indicated that encoding and retrieval happened (almost) concurrently, because the word recognition responses were very fast (R–E+ = 871 ± 13 ms; R–E– = 862 ± 11 ms; R+E+ = 878 ± 13 ms; R+E– = 872 ± 7 ms). Importantly, none of the four trial types showed any significant difference in RT (all *p* > 0.10). Thus, exposure time to the spatial scenes was identical for all conditions and cannot account for the difference in subsequent memory performance.

#### Competition between scene encoding and word retrieval.

To asses our prediction regarding a competition between successful encoding and retrieval, we calculated separate d-primes for the spatial scenes depending on whether the accompanying word was correctly recognized (R+) or not (R–).

Since new scenes were never coupled with words and, therefore, could not be assigned to either of the two conditions, we assumed a constant false-alarm rate for new scenes for both conditions. Confirming our prediction, the d-prime for scene recognition was significantly lower (*t* = 4.08, *p* = 0.0035) when a word was simultaneously recognized (0.63 ± 0.07), as compared to when a word was forgotten (0.76 ± 0.07; Figure 2A). Importantly, the similar RTs across conditions indicate that the competition between encoding and retrieval is not simply due to general effort or interference from perceptual or motor processes.

**Figure 2:**
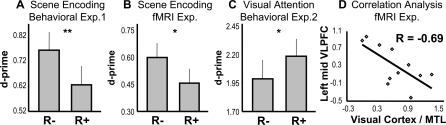
Behavioral Results and fMRI Correlation Analysis Bars showing d-primes for scene encoding or target detection during concurrent recognition hits (R+) and misses (R–) for *Behavioral Experiment 1* (A), *fMRI Experiment* (B), and *Behavioral Experiment 2* (C). Note: * *p*-value < 0.05 and ** *p*-value < 0.005, and the error bars represent SEM. (D) Scatterplot showing the correlation between activity in left mid-VLPFC and the encoding suppression in the visual cortex/MTL. Each point represents the data from a single participant.

### fMRI Experiment: Word Retrieval and Scene Encoding

#### Behavior.

Overall memory performance as defined by d-prime was 0.90 ± 0.10 for the word recognition and 0.60 ± 0.08 for recognition of the spatial scenes. Again, participants used a conservative response criterion as indicated by the response bias criterion C for word retrieval (C = 0.59 ± 0.17; *t* = 3.41, *p* = 0.0067) and for scene retrieval (C = 1.40 ± 0.14; *t* = 9.78, *p* < 0.0001). Replicating our finding in *Behavioral Experiment 1*, we again found that the d-prime for scene recognition was significantly lower (*t* = 2.34, *p* = 0.041) when a word was simultaneously recognized (0.46 ± 0.08), compared with when a word was forgotten (0.60 ± 0.08; Figure 2B). Also comparable to *Behavioral Experiment 1*, RTs during the word recognition task were similar (mean RTs: R–E+ = 861 ± 19 ms; R–E– = 848 ± 15 ms; R+E+ = 859 ± 26 ms; R+E– = 850 ± 19 ms). Again, none of the four trial types showed any significant difference in RT (all *p* > 0.10). Thus again, exposure times for the spatial scenes were identical and cannot account for the difference in memory performance or for any of the fMRI results.

#### Encoding-retrieval interaction in PCC.

In line with previous fMRI studies, we predicted that PCC would show activity selectively associated with successful retrieval. Confirming this prediction, the PCC (Montreal Neurological Institute (MNI) coordinates [*x*,*y*,*z*] = −6, −27, 21) showed significantly greater activity (*t* = 4.39) during R+E– than R–E– trials. We also predicted that PCC activity would show an interaction between outcome (successful versus unsuccessful) and stage (encoding versus retrieval). To test this prediction, we combined the PCC activity for the four different trial types according to stage and outcome. Thus, we coded unsuccessful encoding as E– = [R–E– and R+E–], successful encoding as E+ = [R–E+ and R+E+], unsuccessful retrieval as R– = [R–E– and R–E+], and finally, successful retrieval as R+ = [R+E– and R+E+]. Next, we conducted a stage (encoding/retrieval) × outcome (successful/unsuccessful) repeated measures analysis of variance (ANOVA). As illustrated in Figure 3A, the results support a competition between encoding and retrieval processes in PCC by indicating a significant interaction between outcome and phase (F,1,10) = 12.2, *p* = 0.0058) reflecting the highest level of PCC activity during successful retrieval and unsuccessful encoding, and the lowest level of activity during unsuccessful retrieval and successful encoding.

**Figure 3:**
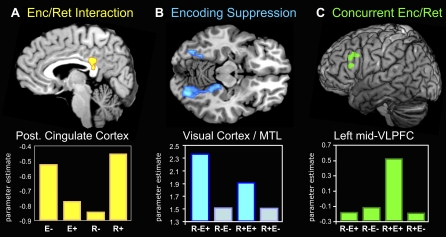
fMRI Results For the purpose of illustration, all activations are shown at *p* < 0.005. (A) Interaction between stage (E/R) and outcome (+/-) in PCC, E– = (R–E– and R+E–), E+ = (R–E+ and R+E+), R– = (R–E– and R–E+), R+ = (R+E– and R+E+). (B) A thre-step analysis indicated encoding suppression in the visual cortex and medial temporal lobe during concurrent retrieval. In step 1, a “localizer” task identified regions related to scene processing. In step 2, we defined encoding success areas within the remaining regions using the contrast R–E+ > R–E–. In step 3, we tested whether the mean encoding success activity of the remaining regions was reduced when successful retrieval happened concurrently. (C) Greater activity in left mid-VLPFC during concurrent, as compared to separate, successful encoding and retrieval (R+E+ > R+E– and R–E+). Bar graphs indicate the mean of all activated voxels (*p* < 0.001, uncorrected).

#### Suppression of successful encoding activity.

Regarding the neural consequences of concurrent encoding and retrieval, we predicted that successful retrieval should by accompanied by a suppression of successful encoding activity. To test this prediction, we first identified the regions that showed greater activity during scenes that were subsequently remembered (R–E+) than scenes subsequently forgotten (R–E–), and that were also associated with perceptual scene processing as indicated by a “localizer” task (see Materials and Methods). In line with previous studies of scene encoding [10–13], these regions included the visual cortex and eareas within MTL (maxima in MNI [*x*,*y*,*z*]: left visual cortex = −30, −57, −9; *t* = 7.84; right visual cortex = 36, −72, 15; *t* = 9.06; left posterior parahippocampal cortex = −33, −37, −15; *t* = 6.48; right posterior parahippocampal cortex = 30, −33, −21; *t* = 7.55; Figure 3B). Subsequently, we tested whether the overall activity of all the significant voxels was reduced when successful retrieval happened concurrently. The results confirmed our prediction; areas associated with successful encoding showed a significant suppression of activity when retrieval succeeded simultaneously (R+E+ < R–E+: *t* = 2.97, *p* = 0.014, Figure 3B). We also addressed a possible concern that the encoding success regions we selected might be biased toward R–E+ trials, because they were identified based on the contrast between R–E+ and R–E– trials. Yet, when we performed the same analysis based on an unbiased encoding success contrast including all trials (R+E+ and R–E+ > R+E– and R–E– at *p* < 0.001, uncorrected), we found a similar difference (R+E+ < R–E+: *t* = 2.45, *p* = 0.034).

#### A role for the left mid–ventrolateral prefrontal cortex in resolving the memory competition.

Yet, despite the apparent competition between encoding and retrieval, on several trials, people were actually able to both remember the word and learn the spatial scene. Thus, we proceeded to investigate whether there might be a specific brain region that plays a role in performing encoding and retrieval at the same time. Following previous findings, a possible candidate would be the prefrontal cortex, since this region has been associated with cognitive control [14] and top-down attentional selection of visual information [15]. To investigate this prediction, we compared trials in which both encoding and retrieval were successful with trials in which only one of these processes was successful (R+E+ > [R+E– and R–E+] at *p* < 0.001, uncorrected). As shown in Figure 3C, we found a region within the left mid-ventrolateral prefrontal cortex (mid-VLPFC ; BA 45), which was activated during R+E+ trials (MNI [*x*,*y*,*z*] = −45, 27, 15; *t* = 4.93), but not during R+E– and R–E+ trials.

Given the involvement of left mid-VLPFC in simultaneous encoding and retrieval (R+E+ trials), we next investigated whether this region might help to resolve the encoding/retrieval competition. To test this idea, we calculated the correlation between *concurrent encoding/retrieval* activity (R+E+ > [R+E– and R–E+]) in left mid-VLPFC and the amount of *encoding suppression* (R–E+ > R+E+) in the visual cortex/MTL. The results revealed a significant negative correlation (Figure 2D, *R* = −0.69, *p* = 0.019), indicating that more activity in the left mid-VLPFC is accompanied with a smaller difference in visual cortex/MTL activity between R–E+ and R+E+ trials. Thus, the fact that left mid-VLPFC is exclusively active for R+E+ trials and, at the same time, is associated with less encoding suppression, indicates that this region plays an active role in resolving the competition between learning and remembering.

### Behavioral Experiment 2: Word Retrieval and Visual Attention

We also addressed a critical concern: despite the fact that the response times for the four critical trial types did not differ, one could still argue that retrieval success results in greater attentional capture than retrieval failure, which in turn could account for the observed reduction in scene encoding. To address this important issue, we conducted a follow-up behavioral experiment in which we replaced the scene encoding with a visual attention task (see Materials and Methods and Figure 4). Memory for the words as defined by d-prime (1.01 ± 0.08) was similar to the previous experiments (*Behavioral Experiment 1*: *t* = 0.33, *p* = 0.75; *fMRI Experiment*: *t* = 0.92, *p* = 0.38). D-prime was also used as a measure of visual attention, as assessed by the detection of a small target concurrent with word recognition. Overall, all participants showed clear evidence of successful target detection (d-prime = 2.07 ± 0.63), and again, they used a conservative response criterion for both word retrieval (C = 0.31 ± 0.11; *t* = 2.78, *p* = 0.019) and visual attention (C = 1.09 ± 0.13; *t* = 8.23, *p* < 0.0001). Mean reaction times for the four critical trial types, which in this case combined word recognition (hits = R+/ misses = R–) with target detection (hits = T+ / misses = T–) were 892 ± 17 ms for R–T+, 872 ± 20 ms for R–T–, 838 ± 18 ms for R+T+, and 843 ± 15 ms for R+T–. Although a two-sample t-test indicated that these reaction times were comparable to the ones in *Behavioral Experiment 1* and the *fMRI Experiment* (all *p* > 0.10), a within-group paired t-test indicated a significant difference between R–T+ and R+T+ (*t* = 2.36, *p* = 0.037), and a trend between R–eT+ and R+T– (*t* = 2.13, *p* = 0.056). Although we did not find this in the first two experiments, slower reaction times for misses than for hits are a common finding in memory studies, and are taken to reflect a more demanding and extended search process [16].

**Figure 4:**
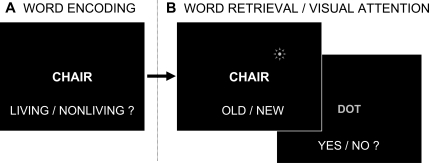
Visual Attention Task The task involved two phases: a *word encoding phase* and a *word retrieval/visual attention phase*. During the *word encoding phase* (A), participants studied words while making semantic (living/nonliving) decisions about the study items. During *word retrieval/visual attention phase* (B), participants performed an old/new word recognition task including old words presented at word encoding as well as new words that were not seen at study. On half of the trials, a small dot was flashed in the background during word recognition. After every recognition response, a second response was required to indicate visual detection of the target dot.

Importantly, in order to assess whether the competition between encoding and retrieval was merely a result of attentional differences between retrieval hits and misses, we calculated the proportion of successfully detected targets depending on whether or not the accompanying word was correctly recognized. In this case, the results actually showed the opposite effect compared with concurrent word retrieval and scene encoding: the d-primes for target detection were significantly higher (*t* = 2.67, *p* = 0.022) when a word was simultaneously remembered (2.18 ± 0.15), compared with when a word was forgotten (1.99 ± 0.16; see Figure 2C). Thus, these findings indicate that retrieval misses actually capture more visual attention than hits, and consequently, that an attentional explanation cannot easily account for the competition between learning and remembering, which was observed in *Behavioral Experiment 1* and the *fMRI Experiment*.

## Discussion

Using a novel paradigm that forces encoding and retrieval to happen within a brief period of time (Figure 1), we provide evidence for a competition within our memory system between learning and remembering. We also provide evidence indicating a possible role for mid-VLPFC in resolving the memory competition. Finally, we show that the memory competition cannot merely be explained by an attentional account.

The rationale for this study was derived from recent observations indicating opposite levels of activity in PCC during successful encoding and retrieval [4–8]. We confirmed these cross-experiment observations by showing an interaction between encoding- and retrieval-related activity in the PCC. As shown in Figure 3A, this interaction reflected less PCC activity for E+ than E– trials, but more activity for R+ than R– trials. To our knowledge, this is the first study to demonstrate the opposite involvement of PCC in encoding and retrieval within the same experiment, subjects, and trials.

We also report a new memory effect: learning (successful encoding) and remembering (successful retrieval) compete when both processes happen within a brief period of time (Figure 2A). We replicated this finding in a subsequent fMRI study (Figure 2B), which also revealed a neural correlate of the behavioral memory effect: successful encoding activity in visual cortex and medial temporal lobe was suppressed when concurrent retrieval was successful (Figure 3B). Further study of the specific circumstances under which this memory effect takes place is still required. For instance, it remains unclear what the temporal order (does retrieval affect encoding or vice versa?) and time window of successful encoding and retrieval processes should be, for the interference to occur.

Interestingly, there is other behavioral evidence indicating that retrieval can induce forgetting [17–19]. Learned information tends to be forgotten when it is semantically related to other information that is rehearsed by means of repeated retrieval. Such retrieval-induced forgetting is thought to be the result of inhibitory control processes that reduce semantic interference by suppressing competing memory traces [17–19]. Yet, in retrieval-induced forgetting paradigms, the negative effect of retrieval involves old memories that have already been stored, whereas here, it involves the concurrent encoding of novel information. Thus, in general, the current findings and those obtained in retrieval-induced forgetting paradigms cannot be easily compared.

Despite the encoding/retrieval competition, on several trials, all participants were actually able to both remember and learn. Follow-up fMRI analyses showed that these trials were accompanied by selective activity in the left mid-VLPFC (Figure 3C). A subsequent correlation analysis indicated a negative relationship showing that more activity in left mid-VLPFC was coupled with less encoding suppression. Together, these findings suggest a role for the left mid-VLPFC in resolving the competition between learning and remembering. Given that encoding and retrieval were forced to occur within a brief period of time, we propose that the role of left mid-VLPFC involves the facilitation of rapid switching between the encoding and retrieval processes.

A role of left mid-VLPFC in rapid memory switching fits well with evidence implicating this region in flexible behavior and cognitive control. Outside the domain of memory, several studies have linked left mid-VLPFC activity to situations requiring flexible switching between different task sets or rules. For example, a recent fMRI study showed that activity in left mid-VLPFC is linked to task-switching [20]. In this study, people performed two semantic classification tasks (large/small or man-made/natural). When a task-switch was required, trial-by-trial fluctuations of left mid-VLPFC activity were associated with faster responses, while right frontal activity was associated with a sustained increase in reaction times (independent of the task). Based on these results, the authors concluded that the left mid-VLPFC is associated with rapid and efficient task-switching. Complementing these fMRI data, a recent clinical study reported that patients with damage to mid-VLPFC show substantial impairments when rules are switched during an oculomotor task [21]. Within the domain of memory, a recent review associated left mid-VLPFC (BA 45) specifically with a post-retrieval selection process, which operates to resolve conflict among retrieved representations [22]. This idea is based on the finding that this region shows greater activity with increasing numbers, or strength, of retrieved competitors [23,24]. Here, we confirm that left mid-VLPFC shows greatest activity in situations where conflict is largest (R+E+). Yet, the current study extends these findings in an important way. First, we show that left mid-VLPFC activity is not only associated with competition during retrieval, but also, with the conflict that arises when retrieval is competing for resources with concurrent encoding. Second, by showing a negative coupling between left mid-VLPFC activity and the encoding suppression effect in the visual cortex and MTL, we provide new evidence that this region is not merely associated with high-conflict memory conditions, but actually aids in resolving conflict.

Finally, we addressed a crucial issue regarding the possible role of attention in the competition between learning and remembering. Despite the fact that the response times for the four critical trial types did not differ, one could still argue that retrieval success results in greater attentional capture than retrieval failure. This aspect, in turn, could account for the observed reduction in scene encoding. Yet, the results of *Behavioral Experiment 2* (Figure 2C) contradict this explanation. In fact, when the scene-encoding task was replaced with a visual attention task the retrieval effect showed a reversal: the chance of detecting the target dot was significantly smaller when retrieval failed (R–) than when retrieval succeeded (R+). Hence, these results indicate that retrieval failure is actually accompanied by greater engagement of selective attention than retrieval success.

Overall, this study not only provides novel insight into our capacity to learn and remember, but also increases our general understanding of the neural mechanisms underlying flexible behavior. Virtually all interactive situations we encounter in our daily lives require rapid switching between learning and remembering. For example, normal social communication requires that we process the new information another person is providing. While listening, we are already retrieving information in preparation of an appropriate reply. Other every-day examples are driving through an unfamiliar city while rapidly interpreting familiar traffic signs, and encountering various store products during shopping while remembering what we need. In this respect, it is interesting to note that conditions that compromise mid-VLPFC function, such as normal aging [25], are also associated with impairments in these every-day activities.

On a final note, although the opposite levels of activity in PCC during encoding and retrieval formed the rationale of the study, it should be mentioned that, given the relatively low spatial resolution of fMRI, one should be careful when interpreting this finding in terms of a neural bottleneck regarding encoding and retrieval processes. A single voxel can contain thousands of neurons, some of which can increase in activity while others decrease. These changes could easily sum to zero in terms of fMRI signal. Thus, while the overall signal within a voxel cannot increase and decrease at the same time, fMRI does not allow determining whether different neural signals within the voxel are simultaneously increasing and decreasing. Also, we should state that we are not claiming that encoding and retrieval are fundamentally distinct processes that always compete. Actually, according to transfer-appropriate processing [26] and reactivation accounts [2,27], the overlap between encoding and retrieval processes forms the most important determinant of memory performance. The present data merely indicate that encoding and retrieval compete for neural resources when these processes are forced to occur within a brief period of time and involve different sources of information.

In conclusion, the present study yielded five main findings. First, we confirmed and extended previous evidence indicating opposing levels of activity in PCC during learning and remembering. Second, in line with a competition in our memory system, we report a new memory effect: successful retrieval has a detrimental effect on memory encoding when both processes happen within a brief period of time. Third, we found that this behavioral effect is coupled with suppression of encoding-related brain activity. Fourth, we identified a region within left mid-VLPFC that was negatively correlated with the encoding suppression effect. This finding suggests that this region may facilitate rapid switching between encoding and retrieval processes. Finally, a follow-up behavioral study provided indications that the competition between learning and remembering is not due to attention, but truly reflects a memory phenomenon. More generally, these findings show that, although learning and remembering compete, there are certain conditions in which this bottleneck in our memory system can be resolved.

## Materials and Methods

### Behavioral Experiment 1: Word retrieval and scene encoding.

*Participants.* Nine participants (five female), with a mean age of 24 years, were recruited from the University of Amsterdam to participate in this experiment. Participants were right-handed, native Dutch speakers with no history of neurological problems and were paid EUR€20 for participation. Participants gave their informed consent and the study met all criteria for approval of the Academic Medical Center Medical Ethical Committee.

*Stimuli.* The stimulus material consisted of 600 words and 720 spatial scenes. All words were nouns selected from the MRC Psycholinguistic database (http://www.psy.uwa.edu.au/mrcdatabase/uwa_mrc.htm) and subsequently translated to Dutch. Words ranged from five to 11 letters long and were of moderate frequency. The spatial scenes consisted of colorful bitmap images (color: 24-bit, resolution: 500 × 375, format: BMP). All images displayed outdoor or indoor scenes with a spatial environment and were selected from an internet database (http://www.flickr.com). Stimuli were generated by a Pentium PC and presented using E-Prime software (Psychology Tools Inc.). The images were displayed on a computer monitor, and responses were collected via a keyboard.

*Procedure.* The behavioral task was designed with the intention to be applied within the context of an fMRI study at a later stage (see *fMRI Experiment.*). The task consisted of three phases, a *word encoding phase* (Figure 1A), a *word retrieval/scene encoding phase* (Figure 1B), and a *scene retrieval phase* (Figure 1C).

During the *word encoding phase*, participants studied 500 words presented on a computer screen while making semantic decisions about the study items (living versus nonliving). Responses were made via a button-press with the right hand, and after the participant responded, the stimulus was instantly removed. The duration of each trial was 1,200 ms., and the inter-trial-interval (ITI) lasted 500 ms. Participants were uninformed that they would later be asked to recall the words during the subsequent *word retrieval/scene encoding phase*.

During the *word retrieval/scene enceoding phase*, participants performed a word recognition task. The words consisted of the items previously presented during the *word encoding phase* (old words), as well as words that were not seen at study (new words). The key difference with a standard old/new word recognition test is that, while recognizing the words, participants incidentally encoded spatial scenes presented in the background. Similar to the *word encoding phase*, participants were uninformed that they would later be asked to recall the spatial scenes. Specifically, participants were told that the images were merely there to provide a memory context, that they should focus on the word recognition task, and that they should respond as quickly and accurately as possible. Signifying the effectiveness of our instructions, participants all indicated that they did not anticipate a subsequent retrieval test including the background scenes. In order to ensure a stable contrast between words and scenes, the words were presented on top of a small rectangle overlaying the center of the background image. Participants performed 600 recognition trials, which lasted maximally 1,200 ms. After the participant responded, the stimuli were instantly removed and followed by a 100-ms visual mask containing Gaussian noise to prevent additional visual processing. The ITI varied between 500–3,200 ms. During the *scene retrieval phase*, memory for the spatial scenes encoded during the previous phase was tested with a scene recognition task. Presentation of the scenes was self-paced and occurred on the same computer as used in the *word encoding phase*.

During both word and scene retrieval, we presented five times more “old” than “new” items (words 500 old versus 100 new; scenes 600 old versus 120 new). There were two reasons for the high number of old relative to new items. First, initial pilot studies had indicated that concurrent scene encoding was very difficult, resulting in a relatively low number of remembered scenes. Second, we were only interested in retrieval of old items and not in classification of new items. Thus, we presented more old items to ensure a sufficient number of events for each of the four trial types in a future fMRI experiment. Because the skewed distribution of old and new items could potentially lead to a liberal response bias towards old items, we instructed participants to make an “old” response only when they were absolutely certain. Confirming that participants followed our instructions appropriately, a response bias analysis (C = −0.5 × (*Z*_FA-rate_ + *Z*_HIT-rate_)) [9] showed that they used a generally conservative response criterion (see Results). After finishing the experiment, we questioned the participants regarding their experience. None of the participants reported that he or she was aware of the high number of old items.

### fMRI Experiment: Word retrieval and scene encoding.

*Participants.* Fifteen additional participants (seven female) recruited from the University of Amsterdam with a mean age of 24 participated in this experiment. Again, all participants were right-handed, native Dutch speakers with no history of neurological problems and were paid EUR€40 for participation. Four participants were excluded based on an extremely low memory performance (d-prime < 0.20 for spatial scenes).

*Stimuli.* The stimulus material consisted of the same 600 words and 720 spatial scenes as used in *Behavioral Experiment 1*. The experimental task was also identical to the previous experiment (Figure 1A), except that the *word retrieval/scene encoding phase* was performed inside the MRI scanner.

“*Localizer*” *task.* Preceding the *word retrieval/scene encoding phase*, participants were presented in the scanner with a “localizer” task involving passive viewing of either 80 spatial scenes, 80 four-letter words, or a fixation cross. These stimuli were distributed over 12 30-s blocks (scenes-words-fixation; scenes-words-fixation; scenes-words-fixation; scenes-words-fixation). The “localizer” task was used to identify brain regions generally involved in scene versuse word processing, and none of the stimuli wase used in any of the other experiments.

*Data acquisition.* fMRI images were collected with a Phillips Intera 3.0T using a standard SENSE head coil and a T2* sensitive gradient echo sequence (96 × 96 matrix, time of repetition (TR) 2,000 ms, echo time (TE) 30 ms., flip angle (FA) 80°, 34 slices, 2.3 mm × 2.3 mm voxel size, 3-mm thick transverse slices). Stimuli were projected on a screen at the front end of the scanner and observed via a mirror mounted on the head coil. The participant's head was fixed by foam and the participants wore earplugs to reduce scanner noise. The behavioral responses were collected by an MR-compatible four-button box (Lumitouch).

*fMRI analysis.* Data from the “localizer” task and the *fMRI Experiment* were analyzed using SPM2 (Statistical Parametric Mapping; http://www.fil.ion.ucl.ac.uk/spm). Time-series were corrected for differences in acquisition time and realigned. The images were spatially normalized using the MNI echo planar imaging (EPI) template included in SPM2 and resliced to a resolution of 3 × 3 × 3 mm. Next, the functional images were spatially smoothed using an 8-mm isotropic Gaussian kernel.

Block-related activity in the “localizer” task was assessed by convolving a boxcar function representing the onsets eand offsets of each block with the hemodynamic response function (HRF). Trial-related activity in the *fMRI Experiment* was assessed by convolving a vector of the onset times of the stimuli with the HRF. The general linear model (GLM), as implemented in SPM2, was used to model the effects of interest as well as other confounding effects (scanner drift and motion). Statistical parametrical maps were identified for each participant by applying linear contrasts to the parameter estimates (beta weight) applying to the events of interest, resulting in a t-statistic for every voxel. Random effects analyses were employed to calculate group effects.

For the *fMRI Experiment*, the events of interest were determined by the performance on the memory tasks. Hits and misses for words during the scan-phase were coded as retrieval hit (R+) and retrieval miss (R–), while hits and misses for the scenes, during the subsequent scene retrieval, were coded as encoding hits (E+) and encoding misses (E–). These responses combined resulted in a sufficient number of events (> 30 events) for each of the four relevant trial types in the fMRI analyses (mean: R–E+ = 31 ± 8; R–E– = 181 ± 21; R+E+ = 37 ± 8; R+E– = 222 ± 26). Although the fMRI analysis focused specifically on the old items, new items and omitted responses were also included in the GLM.

*Encoding-retrieval interaction in PCC.* In order to re-examine previous fMRI studies that consistently found increased activity in PCC for successful as compared to unsuccessful memory retrieval [4,5], we calculated the difference in brain activity between R+E– and R–E– trials (*p* < 0.001, uncorrected). Since no encoding occurred, and only the level of retrieval varied, the resulting difference between these trials can only be attributed to retrieval success.

To examine whether the PCC would show an interaction between stage and outcome, we recombined the PCC activity for the four different trial types to reflect unsuccessful encoding (E– = [R–E– and R+E–]), successful encoding (E+ = [R–E+ and R+E+]), unsuccessful retrieval (R– = [R–E– and R–E+]) and successful retrieval (R+ = [R+E– and R+E+]). Next, we conducted a stage (encoding/retrieval) × outcome (successful/unsuccessful) repeated measures ANOVA based on mean cluster activity in PCC.

*Suppression of successful encoding activity.* To test the prediction that successful retrieval leads to suppression of successful encoding activity, we used a three-step approach. In the first step, we used the “localizer” task to identify regions related to visual processing of scenes vs. those involved in the general processing of words (scenes > words at *p* < 0.001, uncorrected). In step two, we looked within these areas for regions that were reliably associated with encoding success, defined as R–E+ > R–E– (*p* < 0.001, uncorrected). Similar to the previous retrieval analysis, no retrieval occurred for these trials and only the level of encoding varied. Thus, the resulting difference between these trials can only be attributed to encoding success. In the final step, we tested whether the mean encoding success activity of the remaining regions was significantly reduced when successful retrieval happened concurrently.

### Behavioral Experiment 2: Word retrieval and visual attention.

*Participants.* Twelve additional participants (eight female) recruited from the University of Amsterdam, with a mean age of 25, participated in this experiment. Similar to the previous two experiments, all participants were right-handed, native Dutch speakers, reported no history of neurological problems, and were paid EUR€10 for participation.

*Procedures.* The settings for the word recognition task were identical to the previous experiments, except that the scene encoding task was replaced by a visual attention task, and accordingly, there also was no subsequent scene recognition task (see Figure 4). During the word retrieval phase, participants simultaneously performed a visual attention task. On half of the retrieval trials, which were randomly selected, a small target dot (0.5°) appeared (for 13 ms) at a random location 9° from the centre of the screen, sometime between 50–300 ms after word onset. Similar to the previous experiments, participants were told to focus on the recognition task and to make their recognition responses as quickly and accurately as possible. After the recognition response, participants were asked to indicate, without time limit, whether they had just perceived a dot or not. Similar to the previous experiments, we instructed the participants to only respond positive (old or target detected) when they were certain. Response bias-measures [9] confirmed that participants followed our instructions (see Results).

## Abbreviations

ANOVA: analysis of variance
fMRI: functional magnetic resonance imaging
GLM: general linear model
HRF: hemodynamic response function
ITI: inter-trial interval
MNI: Montreal Neurological Institute
MTL: medial temporal lobe
PCC: posterior cingulate cortex
RT: reaction time
SPM: statistical parametric mapping
VLPFC: ventrolateral prefrontal cortex
